# Engaging underrepresented patient groups in specialised treatment – qualitative results from the PROVIDE-C randomised trial on integrated mental health video consultations for depression and anxiety

**DOI:** 10.1186/s12889-025-25235-1

**Published:** 2025-11-06

**Authors:** Selina Müller, Alexa Ritter-von Kramer, Justus Tönnies, Alina Wildenauer, Michel Wensing, Hans Christoph Friederich, Markus W. Haun

**Affiliations:** 1 Department of General Internal Medicine and Psychosomatics, Heidelberg University, Im Neuenheimer Feld 410, Heidelberg, D-69120 Germany; 2 Department of General Practice and Health Services Research, Heidelberg University, Heidelberg, Germany; 3 German Center for Mental Health (DZPG), Partner Site Mannheim-Heidelberg-Ulm, Germany

**Keywords:** Mental health, Primary care, Integrated care, Telemedicine, Implementation, Qualitative research

## Abstract

**Background:**

Mental health specialist video consultations (MHSVC) offer a promising way to address the growing burden of depression and anxiety. However, their acceptance among groups with lower mental health care uptake and limited technology literacy remains underexamined.

**Objective:**

This study explores how underrepresented patients—elderly, rural, and male individuals with depression or anxiety—experience and accept MHSVC after participating in PROVIDE-C, a randomized trial evaluating a five-session MHSVC intervention in primary care.

**Methods:**

A qualitative interview study in rural Germany used inductive content analysis and the Technology Acceptance Model (TAM). TAM suggests that perceived usefulness and ease of use influence technology adoption.

**Results:**

Among 21 PROVIDE-C participants, attitudes toward MHSVC were largely positive. Patients found the intervention useful for therapeutic alliance, symptom relief, and treatment measures, with many preferring continued sessions. Prior mental health care experience and strong primary care relationships increased acceptance. Some patients, already familiar with videoconferencing due to COVID-19, adapted easily, while those with lower technology literacy relied on technical support in primary care to engage with MHSVC for the first time.

**Conclusions:**

Embedding MHSVC in primary care enhances access for patients hesitant about mental health treatment or unfamiliar with digital tools. The PROVIDE model effectively reaches underserved populations, namely elderly, rural patients, improving access to specialized care and reducing depression and anxiety symptoms, as evidenced by its demonstrated effectiveness.

**Trial registration:**

ClinicalTrials.gov, United States National Institutes of Health NCT04316572. Prospectively, registered on 20 March 2020.

**Supplementary Information:**

The online version contains supplementary material available at 10.1186/s12889-025-25235-1.

## Background

Mental health disorders contribute significantly to the global burden of disease, with depression and anxiety—the most common psychological disorders—estimated to have increased by approximately 25% during the COVID-19 pandemic, exacerbating personal and economic challenges worldwide [1]. Specialised mental health care is effective in treating depression and anxiety [2], but access rates differ among patient groups. Mental health services tend to be used more frequently by younger, predominantly female individuals, while help-seeking behaviour is generally lower in men and declines with age [3]. Further, long waiting times, particularly in rural areas, often delay access to care, risking symptom deterioration and chronification [4, 5]. Barriers such as limited mobility and poor infrastructure in rural or suburban areas further contribute to reduced treatment uptake [5, 6].

Leveraging technology to improve access to mental health care is a promising option and telemental health has been shown to be effective [7] and cost-efficient [8] in treating depression and/or anxiety, especially suitable for rural populations. Yet, older and rural adults remain underserved regarding telemental health, compared to the general population [9, 10]. Barriers to telemental healthcare in elderly include digital literacy gaps, sensory or cognitive limitations, lack of social support, or stigma [11–13]. Nevertheless, tailoring telemental health intervention to older adults’ needs by targeted training or technology support can lead to successful engagement and satisfaction [14, 15]. These findings highlight the need for interventions adapted to the specific needs of older adults in rural settings, which is the focus of our study.

Primary care physicians (PCPs), as trusted gatekeepers to the healthcare system, are often the first point of contact for individuals with mental health concerns. However, PCPs frequently face time constraints that hinder adequate diagnosis and treatment [16]. Especially for older people in rural areas and lack of mobility, there is little possibility to visit specialists outside the PCP’s office [17], leaving them underrepresented in and at risk of inequitable access to specialised mental health care [18]. Integrated care models [19], which embed specialised mental health services into primary care, along with advancements in telehealth, offer a promising avenue to expand access irrespective of geographical or logistical barriers [20]. The PROVIDE project (ImPROve cross-sectoral collaboration between primary and psychosocial care through implementing VIDEo consultations in primary care; ClinicalTrials.gov NCT04316572) integrated telemental health into primary care by offering mental health specialist video consultations (MHSVC) directly within PCP practices [21]. The intervention led to small but significant reductions in depression and anxiety symptoms, as well as psychological distress linked to somatic symptoms, highlighting its potential to improve access to specialised care in primary care settings [21].

While existing studies suggest that MHSVC can support therapeutic relationships and symptom relief [22], little is known about the experiences of older male patients from rural areas—groups often underrepresented in digital interventions due to limited technology literacy and a higher risk of digital exclusion [11, 23]. Studies specifically focusing on older adults’ engagement with telemental interventions for depression or anxiety are sparse [24]. Understanding these patients’ perspectives is essential to inform the future development and implementation of telemental health services in primary care.

### Theoretical framework

A widely applied theory that aims at explaining perspectives toward adopting, using and integrating technology in real-life contexts is the Technology Acceptance Model (TAM) [25]. In its original version, the model states that the intention to use a certain technology is dependent on the user’s attitude, which itself is influenced by the perceived ease of use and perceived usefulness of the technology [25]. Both of the latter are influenced by variable contextual factors [25]. Perceived ease of use is defined as “*the extent to which the individual believes using the technology would be free of effort*” [25, p.320]. For the purpose of this study, guided by previous TAM-research that adapted definitions to their field [26], we define perceived usefulness as: *The extent to and mechanisms through which patients perceive that the PROVIDE model (five sessions of MHSVC in the primary care setting) will help improving their health condition.*

As the TAM framework considers contextual factors in real-life contexts to predict behavioural intentions, it suits the purpose of delineating major components for engaging underrepresented groups in mental health care. Moreover, we answer calls to identify novel context factors for technology acceptance and to conduct TAM-research with older people as this group was found underrepresented despite demographic aging [27].

## Methods

### Study design

The study was prospectively registered with ClinicalTrials.gov (NCT04316572) on March 20, 2020. Study design and data analysis of this qualitative interview study were guided by a critical realist view [28], using an abductive theory driven approach. We followed the consolidated criteria for reporting qualitative research (COREQ) guidelines (supplementary file 1) for reporting the study results [29].

### Objectives

Our study aims for inductive in-depth understanding of acceptance of MHSVC models, viewed through the lens of the TAM framework and its four main components by:

1. Understanding how primary care patients—who typically have lower uptake of specialised mental health care and experience depression and/or anxiety—perceive the PROVIDE model regarding contextual factors such as previous experiences within the healthcare system.
2. Exploring notions of usefulness of MHSVC (e.g. for therapy process or symptom development).
3. Examining the ease of use or obstacles in using MHSVC from a patient perspective.
4. Evaluating how MHSVC are accepted and conceptualized (particularly among groups underrepresented in routine mental health care, including older adults, men, and individuals living in rural areas) to assess the intention to use MHSVC in the future.

### Sampling and setting

For this qualitative study we interviewed 21 of 187 patients who had participated in the intervention group of the PROVIDE-C trial in 29 southern German primary care facilities. All patients lived in rural or suburban areas (see Table 2). We applied a stratified sampling approach to capture perspectives often underrepresented in routine mental health care. To this end, we oversampled men (2:1) and aimed for older patients (for practical reasons: men >50 years, women >60 years). Based on guidance for meaning saturation [30] and given that we targeted an underrepresented population also less likely to participate in research, we planned a sample size of at least 16 interviews. We randomly selected and contacted 32 individuals from those meeting the criteria, anticipating a minimum 50% participation rate. In total, 21 completed the final interview.

### Data collection

ARK (Table 1) conducted twenty-one one-off semi-structured interviews (mean 30:07 min; range 13:58–57:07 min) from December 2021 to March 2022 (16 in person, five online via Zoom due to the COVID-19 pandemic). The interview guide (supplementary file 2) was developed by our interdisciplinary research team (Table 1). After an initial formulation of 25 questions relevant to understand perceptions and acceptance of the PROVIDE model and mental health video consultations in a real-life context, the questions were clustered into topics. For each topic, an open main question was developed, supplemented with clarifications, follow-up questions and prompts, to use if needed. The interviewer had no relationship with the interviewees prior to the interviews but explained rationale of the study at the beginning of each interview. Interviews were conducted at the participants’ home. There was no third-party present during the interviews. The interview guide was pilot tested with two patient representatives. No changes to the interview guide were made throughout since it proved to be coherent. All interviews were audio recorded, and field notes were taken; all of which were stored in a password-protected folder at a server of Heidelberg University Hospital. Data saturation was regularly discussed during weekly meetings of the research team.

**Table 1:** Characteristics of the research team members

Name of the team member	Training and expertise	Role in the main trial
Selina Müller (SM)	physician working in psychosomatic medicine; early career researcher	Qualitative data methodology and analysis
Alexa Ritter-von Kramer (ARK)	master’s degree in clinical psychology; worked in counselling at the time of the study	Master’s student in the PROVIDE project, qualitative data aquisition
Justus Tönnies (JT)	PhD, master’s degree in epidemiology	PhD student in the PROVIDE project; trial coordinator
Alina Wildenauer (AW)	Master’s degree in sociology; researcher	Research assistant (coordination, data aquisition)
Michel Wensing (MW)	PhD, master’s degree in sociology, full professor in implementation science	Conceptualization and funding aquisition
Hans Christoph Friederich (HCF)	MD, internal medicine specialist, head of department in psychosomatic medicine, full professor in psychosomatic medicine	Conceptualization and funding aquisition
Markus W. Haun (MWH)	MD, psychologist, internal medicine specialist, attending physician in psychosomatic medicine, senior researcher	Principal investigator of the PROVIDE project

### Data analysis

Audio interviews were transcribed by an external company (Transkripto, Rotterdam, Netherlands). The anonymised transcripts were not returned to participants for comments but a synthesis of results was sent back for member checking [31]. Audio files were destroyed after transcription and familiarization with the data. Members of the research team had access to the transcripts, which have been stored on the password-secured server for analysis until publication or up to ten years after the interview.

**Fig. 1:**
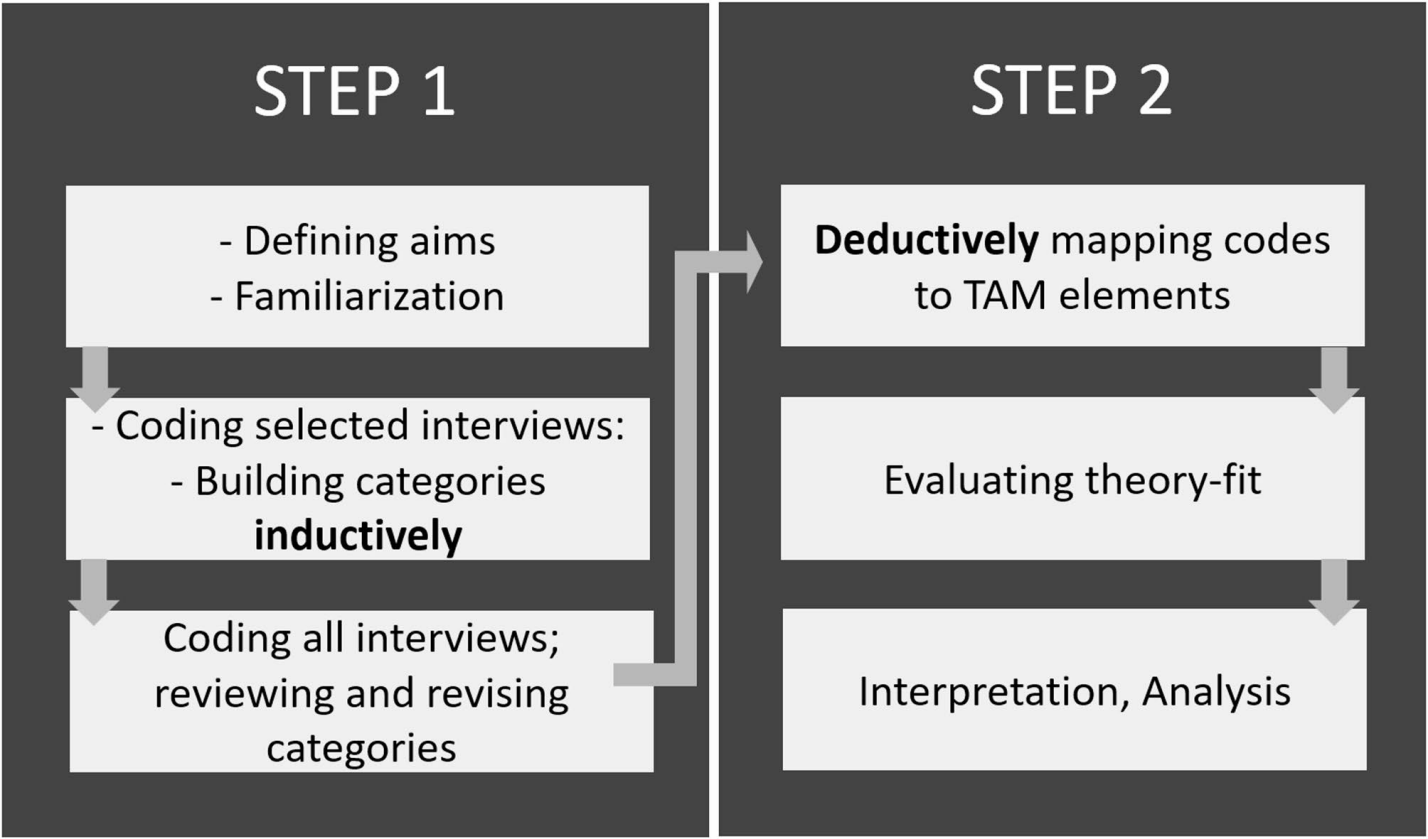
Two-step data analysis process. Step 1: inductive qualitative content analysis [32]. Step 2: deductive mapping of inductively generated codes onto the elements of Theory of Technology Acceptance (TAM) [25]

The data were analysed in two steps (Fig. 1). First, we used inductive category building, guided by qualitative content analysis [32] to identify key themes and subcodes in line with our research questions. After an initial reading and familiarisation with all interviews, four content-rich interviews were selected for computer-assisted, inductive coding using MAXQDA Plus 2022 (VERBI GmbH). Through team meetings and iterative revisions, we developed a coding system comprising ten main categories and 27 subcodes.

In the second step, three team members independently mapped the codes onto the components of the TAM framework using a deductive approach. The resulting mappings were compared and discussed within the team, leading to the final framework presented in the results.

## Results

### Participant characteristics

Of the 32 patients contacted, 21 participated in this qualitative study (Table 2). Six patients did not respond to emails or calls, four declined participation due to lack of interest or scheduling difficulties, and one was hospitalised before providing consent.

When comparing interview participants with those eligible but excluded from the main trial (*N* = 95; 43 declined participation, 52 were lost to follow-up), no significant differences were found in employment status, financial sufficiency, or depressive and anxiety symptom severity (all *P* >.50). Differences emerged for age, gender, marital status (*P* =.003), and education level (*P* =.029), likely reflecting sampling characteristics.

Compared with the remaining intervention participants, interview participants did not differ statistically significant in terms of education level, employment status, financial sufficiency, number of chronic physical diseases, lifetime treatment for depression and/or anxiety, openness to psychotherapy or psychotropic medication, or depressive and anxiety symptom severity (all *P* >.14), apart from age and gender (used as stratification variables), and marital status (*P* =.04). In addition, no significant differences were observed in 12-month follow-up and dropout rates (both *P* >.90), technology commitment (*P* >.90) [33], or consultation setting at home versus PCP-practice (*P* =.78). More information on intervention fidelity is presented in the PROVIDE-C trial publication [21].

**Table 2:** Baseline characteristics of the interview participants

Characteristics	Participants (*n* = 21)
Age (in years)	
Mean (SD)	60 (7)
Median (Min, Max)	60 (50, 76)
Gender (self-identified), *n* (%)	
Female	7 (33)
Male	14 (67)
Marital status, *n *(%)	
In partnership	19 (90.5)
Single	2 (9.5)
Education level, *n*(%)	
No secondary general school-leaving certificate	0 (0)
Secondary general school-leaving certificate	5 (24)
Certificate of ten-grade school of general education in the former GDR/Intermediate school-leaving certificate	5 (24)
Fachhochschule/University entrance qualification	11 (52)
Employment status, *n* (%)	
Employed/self-employed	10 (48)
Retired	4 (19)
On sick leave	4 (19)
Unemployed	2 (10)
Missing	1 (5)
Managing with available income, *n* (%)	
Easily	15 (71)
Not too bad	4 (19)
Difficult some of the time	1 (5)
Difficult all of the time	1 (5)
Degree of urbanization (DEGURBA), *n*(%)	
densely_populated_areas	0 (0)
intermediate_density_area	17 (81)
thinly_populated_areas	4 (19)
Chronic physical disease(s)	
No	7 (33)
Yes	14 (67)
Current psychiatric treatment/psychotherapy, *n* (%)	
No	18 (86)
Yes	3 (14)
Past psychiatric treatment/psychotherapy, *n* (%)	
No	12 (57)
Yes	6 (29)
Missing	3 (14)
Current psychopharmacological treatment, *n* (%)	
No	13 (62)
Yes	8 (38)
Past psychopharmacological treatment, *n* (%)	
No	9 (43)
Yes	3 (14)
Missing	9 (43)
Ever any treatment for depression and/or anxiety prior to trial enrolment, *n* (%)	
Yes	10 (48)
No	11 (52)
Openness to psychotherapy, *n* (%)	
Agree	4 (19)
Strongly agree	17 (81)
Openness to psychopharmacological treatment, *n* (%)	
Strongly disagree	1 (5)
Disagree	5 (24)
Agree	5 (24)
Strongly agree	7 (33)
Declined to answer	3 (14)
Depressive symptom severity (Patient Health Questionnaire 9-item depression scale), mean (SD)	13 (3.8)
Level of depressive symptom severity (Patient Health Questionnaire 9-item depression scale), *n* (%)	
Blank	1 (5)
Mild	3 (14)
Moderate	10(47)
Severe	6 (29)
Highly severe	1 (5)
Level of anxiety symptom severity (Generalized Anxiety Disorder scale, GAD-7), *n *(%)	
Blank	1 (5)
Mild	7 (33)
Moderate	6 (29)
Severe	7 (33)
Lost to follow up at 12 months *n* (%)	
No	17 (81)
Yes	4 (19)
At least one session at home instead of at PCP’s practice (%)	
No	17 (81)
Yes	4 (19)
Technology commitment (mean (SD))	
Technology acceptance	3.5 (0.8)
Technology competence	3.6 (0.9)
Technology control	3.4 (1.0)
Technology commitment (total score)	3.5 (0.8)

### Main results

**Fig. 2:**
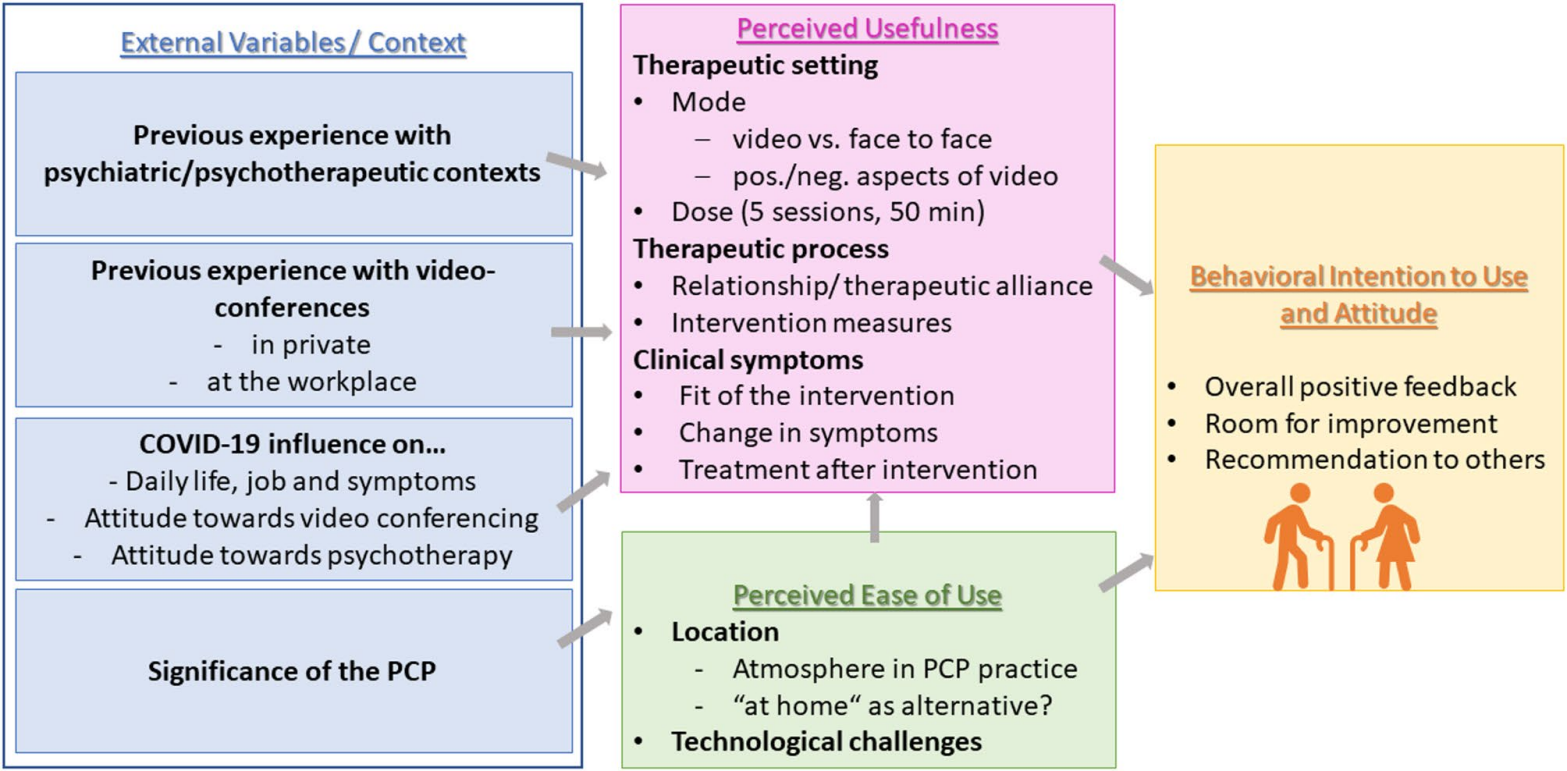
Main results; own illustration based on concepts of the Technology acceptance model (TAM) [25]

Our results are organised according to the four main components of the TAM framework (Fig. 2): external variables (EV), perceived usefulness (PU), perceived ease of use (PEU), and behavioral intention to use (BI). We begin by presenting findings related to EV, followed by a detailed exploration of PU and PEU. The final section focuses on BI, including participants’ attitudes towards using the technology. Corresponding quotes (Q1–Q22) for each theme are provided in Table 3. Patient identifiers include age (in years), gender, and a unique interview number (e.g. 72, male, no. 3).

**Table 3:** Representative quotes by theme. Cross-references for quotes 1-22 (Q1-22) can be found in the results text

**1 External Variables**		
*Q1 *	1.2 Previous experience with psychotherapy	*“I’ve never sought (professional) help. It’s just the way we humans are.” (61*,* female*,* no.13)*
*Q2*	1.3 Previous experience with videoconferencing	*“It was unexplored territory for me. (…) No*,* I was a bit intimidated by it. It’s perhaps a bit of an overcoming.” (68*,* female*,* no.15)*
*Q3* *Q4*	1.4 Influence of COVID-19 on mental health and stigma	*“Nobody trusted each other anymore. (…) People are naturally social beings. (…) Sometimes I was really desperate.” (61*,* female*,* no.15)* *“It’s like: ‘Oh*,* she’s not quite right in the head or something (…) You always get a certain label. (…) But I’m not afraid of the label. You don’t have to be. Because (mental health care) means external support.” (61*,* female*,* no.8)*
Q5 Q6	1.5 Role of the primary care physician	*“I’ve been with him for over thirty years*,* and I think he knows how to tell when I’m in rough shape.” (60*,* female*,* no.2)* *“They know our family*,* my (deceased) husband was also a patient there. They witnessed it all and saw how bad I was at first and how it gradually became clear that I probably wouldn’t be able to cope without help.” (68*,* female*,* no.15)*
**2 Perceived Usefulness**		
Q7	2.1 Therapeutic setting: MHSVC	*“There’s no one with you in the room*,* you can say anything.” (76*,* male*,* no.16)*
Q8	2.2 Therapeutic setting: Dose of 5 sessions	*“I would have liked to have had the option of being able to say (…): ‘I need two or three more (sessions).’ (…) Because you build up a relationship with the person and you’ve already written a piece of history together. And you think through things*,* reflect on possibilities*,* on how to deal with issues and then there is a sudden stop.” (61*,* female*,* no.13)*
Q9	2.3 Therapeutic process: Relationship MHS and patient	*“I could never have imagined that you could establish such good contact with a stranger via the screen and immediately gain so much trust in them.” (61*,* female*,* no.8)*
Q10 Q11	2.4 Therapeutic process: Helpful intervention-methods	*“I realised that there was a plan being followed. Each consultation had a focus topic. It was very structured and helped me to establish a structure.” (56*,* male*,* no.11)* *„So*,* what I really liked was that I wasn’t given so much advice on what to do. Instead*,* situations were described*,* and suggestions were made to think about certain issues. And these then led to what I call self-awareness.” (63*,* male*,* no.19)*
Q12 Q13	2.5 Clinical symptoms	*“Through those five consultations I managed to find a kind of rhythm for myself again. There was also a peek of hope that things could get better. I was really*,* really low. And it was an incredible support for me at the time.” (61*,* female*,* no.13)* *“The other side is how something like this becomes entrenched in everyday life. (…) It takes time (…) in the brain*,* and during this time*,* things can quickly start to slip again.” (63*,* male*,* no.19)*
Q14	2.6 Treatment after intervention	*“But it’s the same everywhere. No matter where you contact and call*,* you get a rejection or no feedback at all. So*,* it always leads nowhere. (…) It’s extremely frustrating*,* I got to say.” (62*,* male*,* no.5)*
**3 Perceived Ease of Use**		
Q15	3.1 Technological aspects: Influence of the medium videoconferences	*“There was a nurse at the PCP’s in charge of technical issues. Difficulties arose once when she wasn’t there; you really needed her to do that.” (61*,* female*,* no.10)*
Q16 Q17 Q18	3.2 Location: Setting in PCP practice vs. “at- home- sessions”	*“Well*,* I’m sixty years old now*,* and I work at a bank*,* but I struggle with all this IT stuff*,* all this crap*,* to put it bluntly. (…) So that was actually quite convenient in the PCP practice.” (59*,* male*,* no.14)* *“I don’t have video at my workplace or at home*,* I don’t have that. I don’t own a camcorder.” (50*,* male*,* no.21)* *“Because I would have had my peace and quiet (at home)*,* I would have been undisturbed. It’s simply a perfect opportunity that you don’t have to drive around.” (63*,* male*,* no.19)*
**4 Behavioural Intention to Use**		
Q19 Q20 Q21	4.1 Overall positive feedback and recommendation to others	*“I think it’s a wonderful*,* marvellous*,* marvellous*,* marvellous thing. I have benefited from it. Endlessly.” (61*,* female*,* no.13)* *“The PCP was informed*,* and a rehabilitation was immediately arranged through him. That was a straightforward concept*,* and I thought that was great because it didn’t go around corners (…). It’s perfect*,* the cooperation was great. (…)I say it was a great advantage*,* because I know how complicated it can be otherwise.” (50*,* male*,* no.21)* *“At first it was a bit like: ‘Gosh*,* how does that work and so on’. Or: ‘What does it look like when I talk to him in the little box (meaning the screen)?’. I was skeptical at first. But it went quite well.” (61*,* male, no.18)*
Q22	4.2 Room for improvement	*“Smoother transition would be nice; if you knew what happens after the five sessions. If both sides agree (on termination)*,* that’s fine. ‘Maybe come back a year or six months later*,* if there’s nothing before then*,* then we’ll do a résumé or a feedback session.’ But if someone realises that they’re just not ready (…) then it feels like a break-off.” (60*,* female*,* no.7)*

### External variables (EV)

#### Previous experience with psychotherapy

While some participants had contacted a psychotherapist, psychiatrist or other form of mental health support (rehabilitation, breathing exercises or mental coaching) at some point in their life, almost half of the participants had never had contact to any of the aforementioned, despite potential need (Q1).

#### Previous experience with videoconferencing

Most participants had used videoconferencing in their jobs or private life in varying intensity, especially since the COVID-19 pandemic, which had helped them to accept the intervention in a video format in the first place. Nevertheless, some explicitly stated that they had never used this type of technology (Q2).

#### Influence of COVID-19 on mental health and stigma

For some participants, the COVID-19 pandemic had no noticeable influence on their mental health as they had perceived social distancing as leading to stress relief and deceleration.

Almost half of the patients (9/21) were certain that the pandemic (e.g. social distancing, fear of catching COVID-19 or increased stress levels at the workplace) negatively influenced their mental health or “*started*” (58, male, no.12) anxiety or depressive symptoms (Q3).

Regarding participants’ openness toward mental health care, only one patient felt that since the pandemic he was “*a tad less afraid*” (56, male, no.11) to start psychotherapy, while most others stated they were generally open. Nevertheless, they revealed stigma around mental health in their social environment. (Q4)

#### Role of the primary care physician

Participants had been invited by their PCP to participate in the study and explained that they were motivated, and did not feel pressured to participate. With strong emphasis, participants shared how they had experienced the intervention as integrated care and how much they valued their PCP’s significant role in health care with long and trusting relationships. (Q5, Q6)

### Perceived usefulness

#### Therapeutic setting: intervention mode MHSVC

For most participants, the medium video was “no disturbing factor” (56, male, no.11) for trust or therapeutic alliance. Some would have preferred face-to-face consultations as they suspected they would have talked more openly about intimate details; still no one had the feeling to have omitted topics for this reason. Others (4/21) considered the video setting a “minimum distance in a positive sense” (61, female, no.13) due to the greater anonymity and a smaller hurdle to talk openly (Q7).

#### Therapeutic setting: dose (5 sessions á 50 min, compared to one session)

While the intervention “was rounded off after the five sessions” (75, male, no.6) for some patients, a majority (16/21) would have opted for more sessions. Some would have liked to talk about their problems in more depth (“The treatment couldn’t even really begin, yet.” (50, male, no.1)), few had the feeling of an abrupt termination and some others would have preferred continued supervision to help with transfer into follow-up treatment. (Q8)

#### Therapeutic process: relationship between MHS and patient

The relationship was generally described positively; participants felt “taken seriously” (60, female, no.7), attributed their MHS as “likeable” (61, female, no.10), “a supporter and guide” (61, female, no.13) who understood their problems. For many (13/21) the positive therapeutic alliance was one reason why they would have liked to continue after five sessions. In few cases (2/21) there was disagreement between participant and MHS that partially affected the relationship. Participants were content though, and often (8/21) they were surprised how fast therapeutic alliance, “trust, an understanding (…), which includes personal matters” (56, male, no.11) could be established (Q9).

#### Therapeutic process: helpful intervention-methods

A central aspect of the therapeutic process for most participants (13/21) was to have a professional person outside their social net who they could talk to, who adequately mirrored their perspective and who they felt understood and validated by. Hearing and being able to accept an alternative perspective on one’s problems was also mentioned as helpful to change one’s own view. Helpful intervention elements were concrete strategies regarding behaviour, social interaction or emotional regulation. Structure within and coherence between the sessions helped perceived usefulness of intervention methods (Q10). Patients valued that they felt invited to self-reflect after the sessions (5/21), to deeper immerse with reasons and solutions for problems (8/21) or to read literature on mental health issues (2/21) (Q11). In general, patients reported that the aforementioned elements helped them to feel more self-confident, either by feeling validated or by receiving strategies that they could apply in the future.

#### Clinical symptoms

A majority (11/21) of patients saw an acute improvement of symptoms during sessions, mentioning for example feeling to have “turned one or two small cogs” (60, female, no.7), to “have stabilised” (61, male, no.18), to be “calmer and relieved” (61, female, no.10), or to “have found myself again” (61, female, no.10). (Q12)

For some (5/12), the intervention also led to a feeling of long-term improvement where they tried to “recall” (68, female, no.15) strategies and advice when needed. Others had the feeling that after several months the effects decreased slightly. (Q13)

#### Treatment after intervention

There were some patients (12/21) for whom a follow-up therapy was seen indicated after the PROVIDE-sessions and who appreciated that support from the PROVIDE-MHS or their PCP resulted in finding an outpatient-therapy slot. Others (6/21) would have appreciated to receive a fast-track treatment option after participating in PROVIDE. Those, a group of five male respondents, who were still searching for an outpatient-therapy slot at the time of the interview, uttered frustration or resignation with being on waitlists or calling therapists in vain (Q14).

### Perceived ease of use

#### Technological aspects: influence of the medium videoconferences

Advantages of videoconferencing were seen in independence of location, and reduced time and costs due to short distances to the PCP’s office (“no long travels and no waiting times” (61, female, no.10)). Further, as support by practice staff was available, technological burden was minor for patients with little technology literacy (Q15). When asked about perceived challenges or disadvantages of videoconferences, less specific answers were given. Regarding ease of use, two participants felt confused to only see parts of the MHS through the tablet monitor, why one of them speculated that a third person might listen in to the conversations. One hearing-impaired patient pointed out that the tablet loudspeaker couldn’t provide enough volume for him to always fully understand the MHS.

#### Location: setting in PCP practice vs. at-home-sessions

The PCP’s office was described as familiar and trusting (11/21); a neutral place, a possibility to have privacy outside their homes. Many (15/21) appreciated the technical support by the practice staff (no waiting time before the scheduled consultations due to good planning, starting the video conferencing devices for participants, being available for questions). Some (8/21) depended on or explicitly preferred doing the MHSVC in their PCP’s office due to a lack of technology literacy or technical equipment at home (Q16, Q17).

Another group of patients viewed the practice rooms more as an improvised and suboptimal setting for therapy sessions, why some (8/21) could imagine doing video-consultations from home due to a more comfortable atmosphere and as they would not have to leave the house (Q18). Four patients had to do at least one session at home instead of the PCP’s practice due to COVID-19.

### Attitudes and intention to use

#### Overall positive feedback and recommendation to others

Most participants provided overall positive feedback on the study and would recommend the PROVIDE model to others in the same situation. Some also revealed advantages of integrated care as realised though PROVIDE (Q19, Q20).

Some participants had been skeptical in the beginning but viewed the model positively in the end (Q21).

#### Room for improvement

When asked for specific recommendations on how to improve the PROVIDE model, 8/21 participants were content with all aspects of the study and had no critical feedback. Some others (8/21) would have opted for an extension of more than five videoconferencing sessions, either as a more flexible model, more tailored to patients’ needs or in terms of symptom severity, or as one follow-up appointment after a longer period, as suggested by 5/21 patients (Q22).

#### Member checking

We received feedback from 42.9% of patients on the results sent out for member checking. All those participants were content with the results and found their perspective well described. The exception was one male participant who had stated in the interviews that gender of the therapist did not matter to him. After participating in the study (where he had been assigned to a female MHS) he had started consulting a male therapist for outpatient psychotherapy; a comparison allowed him to realise that he could open up more easily to female therapists.

## Discussion

### Principal results

This qualitative analysis found the TAM, a framework for technology acceptance [25], to be suitable to evaluate how primary care patients who are usually underrepresented in routine mental health care (older age, male, rural) accept MHSVC in the PROVIDE model. The PROVIDE model (a mental health care model that involves five MHSVC sessions nested in participants’ primary care practice) allowed patients to use MHSVC in a supportive and trusted primary care environment, to successfully establish therapeutic alliance, in many cases resulting in decreased mental health symptoms. Video consultations, for some normalised during the COVID-19 pandemic, were perceived easy to use in the supporting environment of primary care, especially for those with little technology literacy.

### Limitations

Our study provides insights into the experiences and acceptance of MHSVC among patients typically underrepresented in specialised mental health care, with age and gender playing a role. Notably, only half of the participants had prior treatment for depression and/or anxiety, and within this group, just three in ten had received specialised care from a psychiatrist or psychotherapist, which may have positively influenced acceptance of the PROVIDE-C intervention.

The PROVIDE-C sample was largely representative of the German primary care population [21]. In our qualitative sample, a higher proportion of participants were in relationships (compared with those eligible but not enrolled), likely due to stratified sampling targeting older age groups. Apart from this characteristic, we found no evidence for systematic bias, supporting external validity and generalisability. Nevertheless, selection bias cannot be fully ruled out, as certain groups were excluded from the trial by design (e.g. patients with insufficient German language proficiency; pregnant in second or third trimester, acute psychotic symptoms, suicidal ideations; full list in [21]). Further, individual patients might have had reservations that could have hindered trial-participation in the first place; and we were unable to interview any patients who were eligible but ultimately excluded from the trial.

When comparing interviewed participants with the remaining intervention group, potential indicators of engagement, such as 12-month follow-up rates or consultation setting (home vs. PCP office), did not differ, which reduces the risk of selection bias. Further, neither the main trial nor the interviews overrepresented patients with high technology commitment [33]. Still, engagement is a multidimensional construct, and unmeasured variation in motivation or depth of involvement may have shaped the richness and tone of accounts (e.g. more engaged participants providing richer and more positive accounts, less engaged participants briefer or more critical perspectives). Consequently, some response bias cannot be excluded, although fidelity analyses reported in the main trial paper support the robustness of our findings [21].

Social desirability bias may also have influenced positive statements about mental health care. To counter this, we applied member checking and encouraged critical feedback, which yielded constructive input and suggestions for improvement.

Finally, our data were collected during the COVID-19 pandemic, a unique context that likely influenced perspectives on technology acceptance. Telemental health offers increased drastically during the COVID-19 pandemic [23] and many patients in our study reported being more familiar with videoconferencing since the pandemic, highlighting a shift in technology use during the pandemic. Some interview patients had to do the MHSVC from home because they were at high risk for COVID-19, which underlines the impact of the pandemic for an aging population. Importantly, drop-out rates and outcome did not differ from the remaining participants in the intervention group. Nonetheless, the exceptional circumstances of the pandemic may limit transferability, as patient needs and provider readiness to adopt telemedicine might differ outside the pandemic context. Future research should examine the applicability of our findings beyond the pandemic. Given the promising quantitative results of the PROVIDE model, further studies should examine its implementation and uptake across a broader patient population.

### Comparison with prior work

The TAM framework is highly used and appropriate for a wide range of research contexts, including telemedicine [34]. Use of theoretical frameworks when analysing health care interventions aligns with critical realist views [28] and has been recommended for qualitative projects in RCT research as it can guide deeper understanding for underlying mechanisms [35]. As deductively using a pre-defined theory can lead to biased data and forced fit of results, we started our two-step analysis process with more flexible inductive elements and proceeded with theoretical abduction. Since its first description the TAM has been modified and transferred into different fields of health research [26, 27, 36], leading to modifications such as adding a variety of context factors [26]. Previous research recommended to search for new context variables for the TAM [27]. To what extent our qualitatively observed context variables (experience with technology, mental health and the COVID-19 pandemic) account for variability in quantitative studies on technology acceptance remains to be examined.

In this study, we attempted to better understand factors for engaging patients in primary care into MHSVC, focusing on older people, predominantly male, from rural areas as they face several barriers to specialised mental health care, including telemental health offers and therefore often underrepresented in such services [3–5]. Our findings both confirm earlier research and add novel perspectives.

Similar with what other scholars observed, changing living-conditions during the COVID-19 pandemic seemed to normalise videoconferencing in business and private life for many people and previous experience with technology facilitated acceptance of telemental health [37]. Despite this normalization, non-acceptance of telemedicine is correlated with assumptions that in-person care would be of better quality [36]. Especially older people have been found to feel ambivalent towards e-health use in previous research: While they are willing and curious to adopt technology, low technology literacy and mistrust can reduce self-efficacy [38], resulting in less frequent use compared to younger adults [23, 39]. We also found that mainly the younger participants had had previous contact with videoconferencing in work or private life, which had helped them to better accept MHSVC as a therapy option. For those with little technology literacy, technical issues were often mentioned in the interviews as an important issue.

Nevertheless, and in contrast to other studies [10, 12], technology was mainly not perceived as a burden but as easy to use even for those without previous experience, as they felt supported, which underlines that the PROVIDE model was well suited for an aging population with less technological literacy. Especially for older patients without much technology experience, focusing on support and guidance when using technology in primary care [38] can be suggested as a means to promote health equity in older adults. We believe that the PROVIDE model offers such guidance by integrating videoconferencing into primary care so that patients can familiarise themselves with the technology in a supporting environment.

Especially among men, stigma can negatively affect mental health seeking behaviour, leading to symptom aggravation and ultimately to higher suicide rates [40]. We did not intend to compare subgroups within our qualitative sample. Still, we saw especially men struggling with finding outpatient therapy after the intervention, which mirrors the gender gap in psychotherapy, in which men are usually underrepresented [3].

Previous qualitative research using the TAM constructs, revealed context and trust as relevant factors for technology acceptance, which our study transfers into the mental health care sector [41]. Even though still underexamined, the perspective of elderly regarding telemental healthcare from home has been studied previously [11]. Our approach offers a novel perspective by teasing out the advantages of PCP-hosted telemental health care from a patient perspective. Our results highlight the unique role of the PCP as trusted advisor and the relevance of a tight and trusting relationship with one’s PCP in the secure environment of the PCP’s office to improve access and willingness to adopt mental health care. Especially for those who have never used telemental health offers, a trusted, supportive environment and communication with one’s PCP could increase acceptance and remove barriers regarding fear of low-quality therapy or mental health stigma.

Despite specific prompting by the interviewer, many patients did not rely their reaction to the model on specific intervention components in detail but gave general accounts, which is not uncommon in post-therapy (as opposed to post-session) qualitative interviews on psychotherapy processes [42]. Despite an ongoing debate on the topic, a great body of literature supports that common factors of psychotherapy (e.g. therapeutic alliance or empathy) account for a great variance in symptom improvement, compared to therapy school or specific interventions [43]. This could explain why it is often difficult to tease out what exactly compelled patients to agree with a treatment or to improve in their symptoms. In our study (besides therapeutic relationship and the low-threshold offer through primary care), participants especially liked working on specific techniques to problem solving and to have a feeling of structure throughout the sessions. Both are aspects of the problem-solving therapy approach PROVIDE followed [44] and could imply feeling that the intervention empowered patients to be able to gain control over situations through self-efficacy, which is well known to correlate with depression and anxiety [45].

## Conclusions

The PROVIDE model offers primary care physicians (PCPs) a practical tool to increase access to specialised mental health care, particularly for patients who might otherwise remain untreated. Given the trusted and central role of PCPs in healthcare, they are well-positioned to engage individuals hesitant to seek mental health care, as well as those facing barriers such as mobility issues or the inability to use videoconferencing technology at home. Our quantitative findings indicate that most patients experienced symptom improvement following the intervention [21]. However, the PROVIDE model is not designed to replace traditional psychotherapy. Rather, it focuses on diagnostics, initial stabilisation, and, when necessary, referral to more intensive mental health treatment. Some participants expressed a desire for greater flexibility in the number of sessions beyond the standard five-session framework. To address such expectations, we recommend clear communication about the model’s goals and limitations from the outset. Overall, the PROVIDE model presents a low-threshold, accessible, and user-friendly approach to mental health care with the potential to increase mental health equity among aging adults. By alleviating symptoms of depression and anxiety in a timely manner, it can contribute to reducing the disease burden for individuals with mental health conditions.

## Supplementary Information

Supplementary Material 1.

Supplementary Material 2.

Supplementary Material 3.

## Data Availability

The datasets used and/or analysed during the current study are available from the corresponding author on reasonable request.

## Abbreviations

PCP: Primary care physician
MHS: Mental health specialist
MHSVC: Mental health specialist video consultation
TAM: Technology Acceptance Model
EV: External variables
PU: Perceived usefulness
PEU: Perceived ease of use
BI: Behavioural intention to use
