# Supplementary material for: Engaging underrepresented patient groups in specialised treatment – qualitative results from the PROVIDE-C randomised trial on integrated mental health video consultations for depression and anxiety
Source: BMC Public Health. 2025 Nov 6;25:3817. doi: 10.1186/s12889-025-25235-1 (PMC12590799; doi:10.1186/s12889-025-25235-1)
Supplement: Supplementary file 1 — Supplementary Material 1. [file 12889_2025_25235_MOESM1_ESM.pdf]

Introduction

First of all, I would like to thank you for agreeing to do this interview and for your time. This interview is about your experiences with psychotherapeutic video-consultations in your primary care practice. Other patients could also benefit from this model and your experiences can help to improve the model for future patients. You have already completed questionnaires, but I know that these are often incomplete and that there are often things you would like to add. In order to do justice to you and your opinion, we are conducting this interview today.

Of course, this interview is voluntary and if there is anything you don't want to say, please just say so directly. However, in order to get a complete picture of your experiences, I would ask you to be as open as possible. If you do not feel comfortable, we can take a break or end the interview early. This will not have any negative consequences or disadvantages for you.

I will record the interview so that it can be written down and analysed afterwards. Your details and answers are all strictly confidential. The interview will be anonymized and your statements will not be attributed to you personally should the results be published within a study. There will also be no direct exchange with either your primary care physician (PCP) or the mental health specialist (MHS) consulted.

How much time do you have for this interview? I assume that the interview will take about 20 to 30 minutes. The interview will be roughly divided into three parts, which we will go through one after the other.

Do you have any questions about the process?

Part 1

1. When was your first video consultation? How long were the intervals between your sessions?

2. Please describe your experiences with video-based consultations in your PCP practice.

- What advantages or disadvantages did they have for you?
- Please describe situations that were successful for you and those that you found difficult.

3. What specific experiences did you have with video meetings, including private life, prior to your participation?

- Quick support is often good; but do you think this type of video consultation has additional advantages?

4. Have you had previous experience with face-to-face psychotherapy?

- If so, which setting do you prefer and why?
- How was it for you that the video sessions took place in your PCP's practice? [Some patients appreciate the familiar atmosphere at their PCP's practice.]
- During the course of the treatment, did you think that you would prefer to conduct the video consultation from home? If so, what were these situations and at what point in time? [Some patients report that it is more convenient or safer from home.]

Part 2

5. What do you think was your own percentage of acceptance of the video consultation? What percentage was it your PCP's request?

6. Do you think psychotherapeutic video consultations are appropriate for your particular situation (your symptoms, level of stress)?

- For which issues was this good for you?
- Where might it not have been appropriate?

7. How would you rate the number (five sessions), duration (50 minutes) and duration of the interval between of the video consultation sessions?

- Sometimes you develop a relationship and then it ends after five sessions – how was it for you?
- Would a single session perhaps have been more suitable for you?

Part 3

8. How was the connection to your MHS established? How did the relationship develop?

- Did the fact that the sessions were conducted via video play a role? [In the case of rather negative reports/doubts: With increasing duration, it can also happen that you become estranged. Was it more the MHS as a person who was problematic or the setting, i.e. the fact that the sessions were conducted via video?]

9. Have your experiences with the video consultations improved the way you cope with your problems?

- In which areas are you coping better, in which not?
- Did you miss anything?
- Would you have liked anything to be different? Is there anything you think could be improved?
- What was particularly helpful for you?
- Have you already recommended this form of video consultation to others or perhaps advised against it? Why?

10. Think back to the last few months in which Covid-19 has played an important role. How has your life situation changed as a result? Has the pandemic changed your symptoms? Has anything changed in your attitude towards therapy? Or in particular with regard to video consultations?

11. Is there anything else you would like to add that is important to you?

Thank you for speaking so openly about your experience and for taking the time for this interview. If you have any further questions, please do not hesitate to contact me.
