# Supplementary material for: Engaging underrepresented patient groups in specialised treatment – qualitative results from the PROVIDE-C randomised trial on integrated mental health video consultations for depression and anxiety
Source: BMC Public Health. 2025 Nov 6;25:3817. doi: 10.1186/s12889-025-25235-1 (PMC12590799; doi:10.1186/s12889-025-25235-1)
Supplement: Supplementary file 3 — Supplementary Material 3. [file 12889_2025_25235_MOESM3_ESM.pdf]

Engaging underrepresented patient groups in specialised treatment

Qualitative results from 21 patients from the PROVIDE-C randomised trial on acceptance of integrated mental health video consultations for depression and anxiety

Background: Risks for underrepresentation in mental health treatment

- older age
- male gender
- rural living

Results: Underrepresented patients accept mental health video consultations in primary care

factors for acceptance

- Good therapeutic **alliance**
- **Technology** support in primary care
- Strong primary care **relationships**
- **Symptom relief**

Needs seen by patients:

- more than five sessions
- more outpatient treatment capacities

Conclusion: Video consultations are a low-threshold, accessible, and user-friendly approach to mental health care. They cannot replace psychotherapy but help enhancing service provision in rural and remote areas, and promote mental health equity in an aging population .

Bundesministerium für Bildung und Forschung

PROVIDE

Müller et al. *BMC Public Health*, 2025.
